# Introduction of Application of Gini Coefficient to Heart Rate Variability Spectrum for Mental Stress Evaluation

**DOI:** 10.5935/abc.20190185

**Published:** 2019-10

**Authors:** Miguel Enrique Sánchez-Hechavarría, Shreya Ghiya, Ramon Carrazana-Escalona, Sergio Cortina-Reyna, Adán Andreu-Heredia, Carlos Acosta-Batista, Nicolás Armando Saá-Muñoz

**Affiliations:** 1 Departamento de Ciencias Básicas y Morfología - Facultad de Medicina - Universidad Católica de la Santísima Concepción, Concepción - Chile; 2 Department of Kinesiology, San Francisco State University, San Francisco - USA; 3 Departamento de Ciencias Basicas Biomédicas - Facultad de Medicina 1 - Universidad de Ciencias Médicas de Santiago de Cuba, Santiago de Cuba - Cuba; 4 Hospital Universitario Calixto García - Universidad de Ciencias Médicas de La Habana, La Habana - Cuba; 5 Centro de Simulación - Departamento de Ciencias Clínicas y Preclínicas - Facultad de Medicina - Universidad Católica de la Santísima Concepción, Concepción - Chile

**Keywords:** Gini Coefficient, Heart, Rate, Stress, Psychological/physiopathology, Action, Spectrum, Parasympathetic Nervous System, Simpathetic Nervous System

## Abstract

**Background:**

The Gini coefficient is a statistical tool generally used by economists to quantify income inequality. However, it can be applied to any kind of data with unequal distribution, including heart rate variability (HRV).

**Objectives:**

To assess the application of the Gini coefficient to measure inequality in power spectral density of RR intervals, and to use this application as a psychophysiological indicator of mental stress.

**Methods:**

Thirteen healthy subjects (19 ± 1.5 years) participated in this study, and their RR intervals were obtained by electrocardiogram during rest (five minutes) and during mental stress (arithmetic challenge; five minutes). These RR intervals were used to obtain the estimates of power spectral densities (PSD). The limits for the PSD bands were defined from 0.15 to 0.40 Hz for high frequency band (HF), from 0.04 to 0.15 Hz for low frequency band (LF), from 0.04 to 0.085 Hz for first low frequency sub-band (LF1) and from 0.085 to 0.15 Hz for second low frequency sub-band (LF2). The spectral Gini coefficient (SpG) was proposed to measure the inequality in the power distribution of the RR intervals in each of above-mentioned HRV bands. SpG from each band was compared with its respective traditional index of HRV during the conditions of rest and mental stress. All the differences were considered statistically significant for p < 0.05.

**Results:**

There was a significant decrease in HF power (p = 0.046), as well as significant increases in heart rate (p = 0.004), LF power (p = 0.033), LF2 power (p = 0.019) and LF/HF (p = 0.002) during mental stress. There was also a significant increase in SpG(LF) (p = 0.009) and SpG(LF2) (p = 0.033) during mental stress. Coefficient of variation showed SpG has more homogeneity compared to the traditional index of HRV during mental stress.

**Conclusions:**

This pilot study suggested that spectral inequality of Heart Rate Variability analyzed using the Gini coefficient seems to be an independent and homogeneous psychophysiological indicator of mental stress. Also, HR, LF/HF, SpG(LF) of HRV are possibly important, reliable and valid indicators of mental stress.

## Introduction

The Gini coefficient is a statistical tool typically used in economics to measure income inequality. However, it can be applied to any data with an unequal distribution, including Heart Rate Variability (HRV). HRV is the spectrum of time interval between successive heartbeats (RR interval) over a specific period. This study proposes a novel application of the Gini coefficient to measure the inequality of the power spectral density of RR intervals.

Physical or mental imbalance caused by noxious stimuli can induce stress to normal homeostasis. If the stress on the system becomes chronic, the sympathetic nervous system stays activated, which can cause physical, psychological, and behavioral abnormalities.^1^ Sympathetic nervous system sensitivity to mental stress increases over time and it can increase the risk of future cardiovascular diseases.^2^

HRV measurement has been adopted as a non-invasive and relatively easy method for objective assessment of the severity of stress.^3^ It is a physiological phenomenon of variation in the time interval between heartbeats (RR interval) and is commonly used as a measure of autonomic nervous system activity.^4-7^ Power spectral density (PSD) describes the transformation of periodic oscillations of the heart-rate signals into different frequencies. This transformation gives numerical values about their relative intensity.^8,9^ Spectral methods produce a decomposition of total variation of a data series into its frequency components, which can be expressed in the form of a spectral density function that depicts spectral power as a function of frequency.^10^

A standard for HRV measurement and interpretation of frequency domain variables was published in 1996, and most subsequent studies are based on it.^4,9^ These traditional HRV indices in frequency domain variables include very low frequency [0.0033-0.04 Hz], HF [0.15-0.4 Hz] and LF [0.04-0.15 Hz]. HF has been linked to the parasympathetic influence on the heart, while LF is modulated by baroreflex activity and has been linked to both sympathetic and parasympathetic activity.^4,6,7,11,12^

The power of traditional HRV indices in different bands changes by increasing or decreasing sympathetic or vagal modulation. However, it is unknown how equally this power in each frequency band is distributed during rest. It is also unknown how this power distribution gets affected with changes in sympathetic or parasympathetic modulation. To our best knowledge, the inequality in power distribution of HRV spectrum has not been measured before.

Therefore, the present study aims 1) to apply the Gini coefficient to power spectral density of HRV to measure the inequality of power distribution of frequency bands; 2) to compare the inequality in power spectrums of HRV signals during rest *versus* under mental stress; 3) to evaluate the Gini coefficient as a psychophysiological indicator of mental stress in comparison to traditional HRV indices.

## Methods

### Study population

A total of 13 healthy subjects (7 females, 6 males), age 19 ± 1.5 years, BMI 22.3 ± 1.3 kg/m^2^, participated in this crossover study. An *a-priori* power analysis found that this number of participants would yield 80% power at an alpha level of 0.05. All the subjects were non-smokers and had no history of heart disease, systemic hypertension or any other disease. Participants did not take any medications, drugs or alcohol for 12 hours preceding the experiment and were advised not to drink any caffeinated beverages on the morning of the study. Prior to participation, subjects signed an informed consent. Study procedures were in accordance with the Declaration of Helsinki and the study protocol was approved by the Ethics Committee of the Medical University of Santiago de Cuba.

Experiments were performed in a quiet environment, between 9 a.m. and 12:30 p.m.. ECGs were taken in a sitting position, during rest and during arithmetic mental stress. After attachment of the electrodes, every subject relaxed for 10 min. ECG recordings were obtained during rest with spontaneous breathing for 5 min. Immediately afterwards, subjects performed a mental arithmetic task for 5 min.^13-15^ The mental arithmetic task is one of the most efficient stimuli for inducing mental stress.^16-18^ Briefly, subjects subtracted 7, starting from 1000. They were instructed to subtract as accurately as possible. For a single subtraction, time allowed was 5s and was signaled by a sound. Subjects said the result aloud and after each answer, subjects received verbal confirmation (“right” or “wrong”). They continued successive subtraction, even when the result was wrong. Aside from verbalization of the answers, subjects did not talk during the mental arithmetic challenge.

### Signal acquisition and processing

A PowerLab Acquisition System 8^®^ (ADInstruments) was used to collect the ECG recordings, with a sampling rate of 1000 Hz. A standard Lead II was used for ECG measurement. The Sabarimalai-Manikandan’s^19^ algorithm was used to detect the QRS complexes in the ECG signal, from which RR intervals were obtained. Pre-processing of RR series data was required before HRV analysis in order to reduce analytic errors. The standard deviation filter with percentage filter, with value of 20% from the previous interval, were used to detect ectopic intervals.^20^ Cubic Spline Replacement was employed to replace ectopic intervals using cubic spline interpolation.^21^ Finally, in other analysis of ECG signals, an ECG-derived Respiration Rate (EDR) was computed from raw ECG throughout the procedure via a built-in algorithm of Kubios HRV Premium^®^ 3.0.2 software. The algorithm examined the alterations in the amplitude of the R-peak caused by chest movements during each respiratory cycle. Under stationary conditions (i.e., short-term registrations), the EDR is considered a reliable index of respiratory rates.^22^ A previous study found a reasonable agreement between EDR and a reference respiratory rate derived from nasal/oral airflow.^23^

### Heart rate variability analysis

Using the algorithm described by Berger,^24^ the RR interval sequence was transformed into temporal RR sequence. Pre-processed temporal 5-min RR series were subjected to spectral analysis using the Welch periodogram method to obtain the estimates of power spectral densities (PSD). A total of 2048 samples (5-min RR series) were subjected to computation through the Welch modified periodogram with a Hann window, using segments of 512 samples and overlapping periods of 256 samples. The limits for the spectral HRV bands were delimited from 0.15 to 0.40 Hz for the HF, from 0.04 to 0.15 Hz for the LF, from 0.04 to 0.085 Hz for the LF1 and from 0.085 to 0.15 Hz for the LF2. Absolute PSD were calculated as the integral of each one-sided quadratic spectrogram in the frequency ranges previously defined.

### Proposed Spectral Gini HRV Indices

The Gini coefficient is typically used by economists to measure income inequality. If the income level of the ith [i = 1, 2. . . *N*] house is *xi*, the Gini coefficient is calculated using the following equation:^25^

$$G\left(x\right)=\frac{\Sigma_{i=1}^{N}\sum_{j=1}^{N}\left|x_{i}-x_{j}\right|}{2N\sum_{i=1}^{N}x_{i}}$$

If the incomes of all houses are equal, that is, *x1 = x2 = ⋅⋅⋅ = xN*, the Gini coefficient becomes 0. Additionally, when only one house has income, that is, *x1 > x2 = ⋅⋅⋅ = xN = 0*, the income inequality is maximal and the Gini coefficient is equal to 1.^25,26^ Kyung-Jin You et al.,^26^ in 2016, have proposed the Gini coefficient to quantify the inequality in the power spectrum in the range of interest (*fL-fH* Hz) in electroencephalography for quantifying the depth of consciousness during anesthesia. Applying this to HRV, if each frequency of the power spectrum of the RR intervals is considered as an individual house and the power of the corresponding frequency is considered as the house income, it would be possible to quantify the spectral inequality in terms of the Gini coefficient. Therefore, the Spectral Gini coefficient (SpG) is expressed as:

$${\mathrm{SpG}}_{\mathrm{fL}-\mathrm{fH}}\mathrm{Hz}=\frac{\Sigma_{i=1}^{N}\sum_{j=1}^{N}\left|X\left(f_{i}\right)-X\left(f_{j}\right)\right|}{2\left(H-L+1\right)\sum_{i=L}^{H}X\left(f_{i}\right)}$$

The SpG can measure the inequality in the spectral powers of the RR intervals in each spectral HRV bands employed.

### Statistical analysis

All values were expressed as Mean (X), Standard Deviation (SD) and Coefficient of Variation (CV %), Median [*] and Interquartile Range [¥]. All differences were considered statistically significant for p < 0.05.

The Wilcoxon Signed-Rank Test (non-parametric test) for two related samples was used to compare rest *versus* mental stress. Effect Size with Gates’ delta was calculated and values above 0.80 were adopted with high magnitude.^27^ In order to verify the association between traditional and Spectral Gini indices of HRV during mental stress and rest, Pearson’s correlation was applied to the data with normal distribution, or Spearman’s correlation, for the ones that did not accept this distribution. The normality of the data was initially determined using the Shapiro-Wilk test. Principal Component Analysis (PCA) is a technique to reduce the dimensionality of data consisting of correlated variables while capturing the bulk of variation present in the data.^28^ There are as many principal components (PCs) as there are original variables. Each PC is a linear combination of the original variables with a set of weights called “loadings”, which reflect the correlations between PCs and original variables. PC1 is the directional vector representing the best fit for data cloud. PC2 is the directional vector orthogonal to PC1 that provides the best fit for residual variability in the data, and so on. PCs are mutually uncorrelated. Effective dimensionality reduction is achieved when the first few (dominant) PCs capture most of the variation present in the data. Useful insights on the interrelationship between original variables can be obtained when the dominant PCs have substantive interpretations. The efficacy of the traditional and Spectral Gini Indices of HRV were defined by the Receiver Operating Characteristic (ROC) curve through Sensitivity, Specificity, Area Under Curve and its respective p value were used with Cutoff Points between rest and mental stress set by Youden Index.

All the statistical and mathematical calculations, as well as the processing of the signals, were performed using the Matlab 2012b software.

## Results

Table 1 describes values of traditional and Spectral Gini Indices of HRV at rest and during mental stress. There was a significant decrease in HF (p = 0.046), a significant increase in the heart rate (p = 0.004), LF/HF (p = 0.002), LF (p = 0.033) and LF2 (p = 0.019) during mental stress, compared to rest. A significant increase in SpG(LF) (p = 0.009) and SpG(LF2) (p = 0.033) was observed. Coefficient of Variation analysis showed that Spectral Gini Indices are more homogeneous than traditional Indices of HRV.

**Table 1:** Traditional and Spectral Gini Indices of Heart Rate Variability during rest and mental stress

	Variables	Rest			Mental Stress			Effect Size Gates' delta	p value
		X [*]	SD [¥]	CV (%)	X [*]	SD [¥]	CV (%)		
HRV Index	HR (bpm)	80.32 [75.5]	10.52 [15.6]	13.09	96.41 [91.4]	11.78 [21.1]	12.22	1.52 Large	0.004
	RMSSD (ms)	47.36 [44.10]	22.95 [26.45]	48.45	33.52 [32.20]	17.98 [27.30]	53.63	0.60 Medium	0.009
	EDR (Hz)	0.24 [0.25]	0.05 [0.06]	20.82	0.21 [0.22]	0.04 [0.08]	23.73	-0,6 Small	0.064
Traditional Indices [Bandwidth]	LF (ms2/Hz) |0.04-0.15 Hz|	844.78 [689.27]	627.95 [789.86]	74.33	1373.44 [1123.02]	1003.01 [1560.15]	73.02	0.84 Medium	0.033
	HF (ms2/Hz) |0.15-0.40 Hz|	1281.96 [986.91]	1429.36 [848.70]	111.49	758.91 [517.83]	691.12 [1001.75]	91.06	-0.36 Small	0.046
	LF1 (ms2/Hz) |0.04-0.085 Hz]	291.79 [283.39]	200.64 [180.87]	68.76	267.57 [235.48]	174.23 [224.52]	65.11	-0.12 Small	0.650
	LF2 (ms2/Hz) |0.085-0.15 Hz|	533.69 [435.59]	421.36 [580.17]	78.95	1086.58 [726.52]	861.88 [1308.89]	79.32	1.31 Large	0.019
	LF/HF (ratio)	1.00 [0.69]	0.88 [0.79]	88.2	2.31 [1.93]	0.93 [1.60]	40.34	1.48 Large	0.002
Gin Spectral Indices [Bandwidth]	SpG(LF) |0.04-0.15 Hz|	0.29 [0.29]	0.06 [0.08]	20.40	0.40 [0.39]	0.10 [0.16]	25.62	1.66 Large	0.009
	SpG(HF) |0.15-0.40 Hz|	0.50 [0.50]	0.08 [0.15]	17.35	0.45 [0.47]	0.09 [0.14]	20.00	-0.54 Small	0.133
	SpG(LF1) |0.04-0.085 Hz|	0.24 [0.21]	0.06 [0.07]	25.70	0.23 [0.22]	0.08 [0.12]	36.86	-0.19 Small	0.382
	SpG(LF2) |0.085-0.15 Hz|	0.28 [0.27]	0.07 [0.12]	26.22	0.35 [0.38]	0.10 [0.16]	29.71	0.85 Medium	0.033

p < 0.05. Mean (X), SD: standard deviation; CV: coefficient of variation; HRV: heart rate variability; HR: heart rate; RMSSD: Root Mean Square of the Successive Differences; EDR: ECG-derived; Respiration Rate; LF: low frequency; HF: high frequency; SpG: spectral; Gini coefficient.

[*] Median

[¥] Interquartile Range.

The correlation values between traditional and Spectral Gini Indices of HRV during rest and mental stress are shown in Table 2. During rest, there were high correlations between the HR and the SpG(LF1) (r = 0.721; p = 0.01) and between SpG(LF) and SpG(LF2) (r=0.829; p = 0.01), good correlations between LF and SpG(LF2) (r = 0.645; 0.05), and between LF2 and SpG(LF2) (r = 0.628; 0.05). During mental stress, there was a good correlation between SpG(LF) and SpG(LF2) (r = 0.682; 0.05).

**Table 2:** Correlations between Traditional and Spectral Gini Indices of HRV during mental stress and rest

HRV Indices	SpG(LF)			SpG(HF)			SpG(LF1)			SpG(LF2)		
	Rest	Mental Stress	Total	Rest	Mental Stress	Total	Rest	Mental Stress	Total	Rest	Mental Stress	Total
HR	0.313	-0.413	0.587‡*	0.306*	-0.140	-0.110	0.566†*	-0.112	,151	-0.025	0.463	0.409
RMSSD	-0.084	0.432	-0.029	-0.153	-0.446	-0.173	0.192*	0.053	,122	-0.216	0.267	-0.084
EDR	-0.177	-0.47	-0.466†	-,031	-0.293	-0.055	-0.330*	-0.037	-0.179	0.010	-0.404	-0.320
LF	0.264*	-0.005	0.296*	-	-	-	-0.335*	0.016	-0.177*	0.593^†*^	0.180	0.333*
HF	-	-	-	-0.192*	0.078	-0.026*	-	-	-	-	-	-
LF/HF	0.220*	-0.039	0.397^†*^	0.253*	-0.038	-0.002*	0.104*	-0.207	-0.008*	0.379*	-0.048	0.387*
LF1	0.258*	-0224	0.017*	-	-	-	-0.319*	-0.054	-0.217*	-	-	-
LF2	0.231*	0.041	0.335*	-	-	-	-	-	-0.158*	0.582^†*^	0.17	,335
SpG(LF)	-	-	-	-	-	-	0.390*	0.153	0.177*	0.829^‡^	0.682^†^	0.698^‡^

† p < 0.05;

‡ p < 0.01;

* Spearman’s correlation, for the HRV indices that did not accept normal distribution in Shapiro-Wilk test.

HRV: heart rate variability; HR: heart rate; RMSSD: Root Mean Square of the Successive Differences; EDR: ECG-derived; Respiration Rate; LF: low frequency; HF: high frequency; SpG: spectral; Gini coefficient.

**Figure 1:**
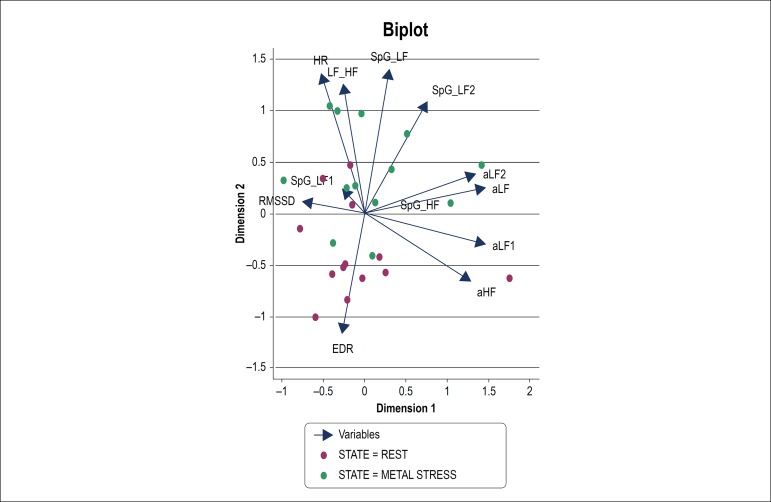
Principal Component Analysis of Traditional and Spectral Gini Indices of Heart Rate Variability during rest and mental stress (aHF = absolute HF; aLF1 = absolute LF1; aLF = absolute LF; aLF2 = absolute LF2). HR: heart rate; RMSSD: Root Mean Square of the Successive Differences; EDR: ECG-derived; Respiration Rate; LF: low frequency; HF: high frequency; SpG: spectral Gini coefficient.

Figure 1 and Table 3 represent Principal Component Analysis (PCA) of Traditional and Spectral Gini Indices of Heart Rate Variability during rest and mental stress.

**Table 3:** Values of Factor Loadings of Traditional and Spectral Gini Indices of Heart Rate Variability during rest and mental stress from Principal Component Analysis

Variable		Dimension 1	Dimension 2
HR		-0.5240	1.3612
RMSSD		-0.7615	0.1257
EDR		-0.2707	-1.1683
LF		1.4742	0.2518
HF		1.2896	-0.6571
LF1		1.4674	-0.2996
LF2		1.3519	0.3851
LF/HF (ratio)		-0.2641	1.2657
SpG(LF)		0.3048	1.4026
SpG(HF)		0.3397	0.0928
SpG(LF1)		-0.2806	0.2438
SpG(LF2)		0.7623	1.0909
Explained variance by		0.3125	0.2578
Total explained variance: 0.5703			

ROC curve and other efficacy values for Traditional and Spectral Gini Indices of Heart Rate Variability are described in Table 4. The cutoff points of the different indicators can be observed in the differentiation of the psychophysiological states obtained from the Youden Index of the ROC curve. Out of the 12 variables studied here, only HR (cutoff point: 83.350 bpm; p = 0.001), LF/HF (cutoff point: 1.02; p = 0.001) and SpG(LF) (cutoff point: 0.356; p = 0.011) show high values of sensitivity, specificity, Youden Index and area under the curve (p < 0.05). HR: heart rate; RMSSD: Root Mean Square of the Successive Differences; EDR: ECG-derived; Respiration Rate; LF: low frequency; HF: high frequency; SpG: spectral Gini coefficient

The PCA helps to reduce the multiple characteristics or variables of a sample to a few dimensions (in this case, only two dimensions). It can be explained as trying to reduce twelve variables of an object to two values or characteristics and to determine which out of these twelve variables are the most robust for those two characteristics (two dimensions), which allow a better study of the object of interest. The important variables for each dimension are those that are higher than 1 or lower than -1. On dimension 1, the variables LF (1.4742), HF (1.2896), LF1 (1.4674) and LF2 (1.3519) have greater weight. On dimension 2, the variables with a greater load are HR (1.3612), LF/HF (1.2657), SpG LF (1.4026) and SpG LF2 (1.0909).

With respect to Figure 1, the relationship between the variables is given by the cosine of the angle formed by each vector representing that specific variable. The more acute the angle, which is to say that it has a tendency to 0, the higher will be correlation, and if the vectors form a 90 degree angle, the variables will not be correlated. On the other hand, if they form an angle of 180 degrees, correlation is inverse. In dimension 2, the vectors of the variables HR, LF/HF and SpG LF form an angle close to 180 with the EDR and therefore, HR, LF/HF and SpG LF are negatively correlated with EDR. The size of the vector is the strength of that variable in that dimension.

## Discussion

The present study aimed 1) to apply the Gini coefficient to power spectral densities of HRV to measure the inequality in distribution of frequency bands; 2) to compare the inequality in power spectrum of HRV signals during rest versus under mental stress; 3) to evaluate the Gini coefficient as a psychophysiological indicator of mental stress in comparison to traditional HRV indices.

In the present study, the traditional indices of HRV during mental stress showed expected results of significant increase in LF power and increase in LF/HF ratio, along with significant decrease in HF power. HRV is a reliable tool to measure psychophysiological stress^29^ and the present results shows significant changes in HRV indices compared to rest.

To the best of our knowledge, this is the first study to apply the Gini coefficient to power spectrums of HRV signal/RR intervals to measure inequality in distribution of power. Conceptually, a Gini coefficient of zero means that the power is distributed equally for all frequencies within a spectral bandwidth. In contrast, a Gini coefficient of 1 suggests that there is a single frequency with the most power within a specific spectral bandwidth, and all other frequencies in the bandwidth have no power. In other words, increase in the Gini coefficient value suggests that there are few frequencies with the most power within that frequency band compared to before. The results showed that there was a significant increase in SpG(LF) during mental stress compared to rest, meaning that during mental stress, not only there was an increase in total power in LF, but also the total power distribution became more unequal and certain frequencies gained the most power. It is noteworthy that the LF2 sub-band (0.085-0.15 Hz) showed increased inequality, as changes in SpG(LF) and SpG(LF2) were significant but not for SpG(LF1) during mental stress. It should also be noted that the traditional HRV index showed a significant decrease in HF power during mental stress, but the decrease in SpG(HF) was not significant. These data suggest that there was decrease in power in the HF band, but the distribution of power within the HF band remained similar during rest and mental stress.

The coefficient of variation showed that, in comparison to traditional HRV indices, Gini spectral indices are homogeneous (see Table 1), meaning that the numeric values of changes in distribution of power during mental stress are located closer to the center (mean) and do not have high SD values like traditional indices. Pearson Correlation (and Spearman’s correlation) Tests revealed poor correlation values between traditional and Spectral Gini Indices during mental stress, even though LF and LF2 of traditional HRV index showed good correlation with SpG (LF2) at rest. This indicated that Gini values are independent of traditional HRV indices and contribute to the additional information not reported until now.

Principal Component Analysis of traditional and Spectral Gini indices helps to reduce the multiple characteristics or variables of a sample (HRV) to a few dimensions (in this case, only two dimensions). It can be explained as trying to reduce twelve variables of an object to two values or characteristics and to determine which out of these twelve variables are the most robust for those two characteristics (two dimensions), which allow a better study of the object of interest. Dimension 2 is what differentiates the state of stress (green arrow on the figure, which tends to go upward) from the state of rest (red arrow on the figure, which tends to go below). Therefore, even though LF and HF have values >1 on Dimension 1, the variables with high load such as HR,LF/HF, SpG LF and SpG LF2 from Dimension 2 are considered physiologically and clinically more important as state indicators.

ROC curve was produced in order to evaluate the efficacy of Spectral Gini indices as an evaluator of mental stress. The cutoff points of the different indicators in the differentiation of the psychophysiological states, obtained from the Youden Index of the ROC curve, can be observed. However, it stands out how the HR (p = 0.001) the LF/HF (p = 0.001) and the SpG (LF) (p = 0.011) constituted the most optimal (ROC model) and effective indicators in the discrimination between rest and mental stress with the best values of sensitivity, specificity, Youden Index and area under the curve (p < 0.05).

The results shown on Table 4 are consistent with the results in Table 1, Figure 1 and Table 3, suggesting that HR; LF/HF and SpG LF were highlighted in the discrimination of the states of rest and stress.

**Table 4:** Efficacy of Traditional and Spectral Gini Indices of Heart Rate Variability in the discrimination of rest and mental stress

Variables	Cutoff point	Sensitivity	Specificity	Youden Index	Area Under Curve	p value
HR	83.350 bpm	1.00	0.769	0.769	0.870	0.001
RMSSD	37.70 ms	0.385	0.307	-0.308	0.325	0.130
EDR	0.2299 Hz	0.385	0.307	-0.308	0.308	0.096
LF	1120.44 ms^2^/Hz	0.538	0.769	0.308	0.651	0.191
HF	623.83 ms^2^/Hz	0.385	0.230	-0.385	0.343	0.174
LF1	239.99 ms^2^/Hz	0.462	0.384	-0.154	0.450	0.663
LF2	581.42 ms^2^/Hz	0.769	0.692	0.462	0.698	0.086
LF/HF (ratio)	1.02	1.00	0.769	0.769	0.870	0.001
SpG(LF)	0.356	0.692	0.923	0.615	0.793	0.011
SpG(HF)	0.505	0.231	0.53846	-0.231	0.373	0.270
SpG(LF1)	0.203	0.538	0.0769	-0.385	0.420	0.489
SpG(LF2)	0.274	0.692	0.615	0.308	0.722	0.054

HR: heart rate; RMSSD: Root Mean Square of the Successive Differences; EDR: ECG-derived; Respiration Rate; LF: low frequency; HF: high frequency; SpG: spectral Gini coefficient.

The significant increase in LF and SpG(LF) power during mental stress allows discussion on the contributing factors for LF power. It is generally accepted that the HF component is a reflex of the parasympathetic activity, and that the LF and LF/HF components are a reflex of both sympathetic and parasympathetic activity.^4^ Breathing rate can influence HRV variables noticeably.^14,30^ Bernardi et al.^14^ have further reported that regardless of the amount of stress involved in the mental task, low breathing rate usually contributes to increase in LF power of HRV. Although there was a decrease in breathing rate during stress compared to rest in the present study, the EDR was 0.21 ± 0.04 Hz or 12.6 ± 0.24 br/min, which is not within the LF components in the RR power spectrum. In other words, in the present study, respiration rate was not responsible for increased LF power during mental stress.

There are few studies examining the contributing factors to LF power of HRV in depth. In their recent study, Roach et al.^31^ reported that 75% of the contribution to LF power comes from fluctuations called ripples, and these ripples are probably due to arterial baroreceptor functions. Reyes del Paso et al.^32^ have showed a strong association between baroreflex activity and mental stress. Vaschillo et al.^33^ have investigated the subdivision of LF in two separate components in young binge drinkers and suggested that these two divisions functionally indicate two distinct physiological parameters. LF1 represents vascular tone baroreflex and LF2 represents heart rate baroreflex activity.

As noted earlier, data analysis from the current study showed increased LF power and decreased HF power during mental stress, along with increased SpG(LF) and SpG(LF2). It is possible that, under stress, a healthy cardiovascular system generates more LF oscillations, especially with power mostly around 0.1Hz frequencies, to regain homeostasis. This possibility is supported by Bates et al.,^34^ who evaluated real-time changes in RR interval spectrum in response to placebo and alcohol. Bates et al.^34^ suggested that under alcohol or other adverse conditions, one of the main adaptations includes maintaining low frequency oscillations even at the expense of high frequency oscillations. This can also explain the lack of changes in SpG(HF) under mental stress. That study also suggested that low frequency oscillations are useful to generate resonance for better adaptation, and 0.1 Hz is one of several resonance frequencies. The current study supports significant increase in LF subdivision during mental stress, and future studies are recommended to investigate the association of 0.1 Hz frequency to arterial baroreflex activity for better understanding of the mechanism of physiological adaptations during mental stress.

## Conclusions

This study successfully applied Gini coefficient to power spectral densities of HRV to measure the inequality in distribution of frequency bands.

These results suggest that during stress (arithmetic challenge), compared to rest, not only total power of low frequency band increases, but the total power distribution becomes more unequal.

Spectral inequalities of heart rate variability analyzed from the Gini coefficient seem to be independent and homogeneous indicators of psychophysiological mental stress compared to traditional indices of HRV as per this pilot study.

Out of traditional and spectral Gini indices of HRV, HR, LF/HF, SpG (LF) seems to be valid and reliable tools as indicators of stress, and this study provides cutoff values for these variables to discriminate the states of stress and rest.

### Study limitations

Among the limitations of this study, the small sample size can be cited. This is a pilot study on Gini coefficient application to HRV spectrum, therefore more studies with larger sample sizes are recommended for better understanding and interpretation of inequalities in power spectral density of RR intervals.

In addition, a mental arithmetic challenge was used to induce mental stress. Although this method is considered valid and reliable, results can possibly be varied under different circumstances, as mental stress is a complex and dynamic phenomenon.

Finally, HRV can be influenced by hormones depending on the menstrual phase in female participants. Although the menstrual phase was not monitored, data for both conditions (rest and mental stress) were collected on the same day in order to minimize baseline variability.
